# Lasting Effects of the Breast and Cervical Cancer Early Detection Program on Breast Cancer Detection and Outcomes, Ohio, 2000–2009

**DOI:** 10.5888/pcd12.140491

**Published:** 2015-07-23

**Authors:** Siran M. Koroukian, Paul M. Bakaki, Xiaozhen Han, Mark Schluchter, Cynthia Owusu, Gregory S. Cooper, Susan A. Flocke

**Affiliations:** Author Affiliations: Paul M. Bakaki, Xiaozhen Han, School of Medicine, and Population Health and Outcomes Research Core, Clinical and Translational Science Collaborative, Case Western Reserve University, Cleveland, Ohio; Mark Schluchter, Susan A. Flocke, School of Medicine and Case Comprehensive Cancer Center, Case Western Reserve University, Cleveland, Ohio; Cynthia Owusu, Gregory S. Cooper, Case Comprehensive Cancer Center and University Hospitals of Cleveland, School of Medicine, Case Western Reserve University, Cleveland, Ohio. Dr Koroukian is also affiliated with Case Comprehensive Cancer Center and Population Health and Outcomes Research Core, Clinical and Translational Science Collaborative, Case Western Reserve University, Cleveland, Ohio.

## Abstract

**Introduction:**

The National Breast and Cervical Cancer Early Detection Program (BCCP) in Ohio provides screening and treatment services for uninsured low-income women aged 40 to 64. Because participation in the BCCP might engender greater self-efficacy for cancer screening, we hypothesized that breast cancer and survival outcomes would be better in BCCP participants who become age-eligible to transition to Medicare than in their low-income non-BCCP counterparts.

**Methods:**

Linking data from the 2000 through 2009 Ohio Cancer Incidence Surveillance System with the BCCP database, Medicare files, Ohio death certificates (through 2010), and the US Census, we identified Medicare beneficiaries who were aged 66 to 74 and diagnosed with incident invasive breast cancer. We compared the following outcomes between BCCP women (n = 93) and low-income non-BCCP women (n = 420): receipt of screening mammography in previous year, advanced-stage disease at diagnosis, timely and standard care, all-cause survival, and cancer survival. We conducted multivariable logistic regression and survival analysis to examine the association between BCCP status and each of the outcomes, adjusting for patient covariates.

**Results:**

Women who participated in the BCCP were nearly twice as likely as low-income non-BCCP women to have undergone screening mammography in the previous year (adjusted odds ratio, 1.77; 95% confidence interval, 1.01–3.09). No significant differences were detected in any other outcomes.

**Conclusion:**

With the exception of screening mammography, the differences in outcomes were not significant, possibly because of the small size of the study population. Future analysis should be directed toward identifying the factors that explain these findings.

## Introduction

Breast cancer is the most common cancer in women, claiming more than 40,000 lives in 2011 alone (1). Of the 232,340 women diagnosed with incident invasive breast cancer in 2013, 42% were aged 65 or older (2).

Although breast cancer is amenable to screening, one-third of breast cancer diagnoses are for regional-stage or distant-stage disease (2), and these patients have a poor prognosis. Numerous initiatives have been developed to increase screening rates by improving outreach and reducing barriers to mammography. One such initiative is the National Breast and Cervical Cancer Early Detection Program (BCCP) of the Centers for Disease Control and Prevention, implemented in 1990 (3). Targeting low-income uninsured women, the BCCP in Ohio provides clinical breast examinations and diagnostic mammography to women aged 40 to 64 and screening mammography to women aged 50 to 64. Outreach efforts have focused not only on one-time mammography use but also on repeat use.

Evaluations of the BCCP have focused mostly on outcomes in women younger than 64 (4–7). However, the benefits of having participated in the BCCP may extend well beyond the years in which a woman is eligible for BCCP services as she becomes age-eligible for and transitions to Medicare. First, past screening behavior (8) is strongly associated with use of subsequent mammography, possibly because of habit and reassurance (9). Second, participation in the BCCP might impart greater knowledge of the benefits of breast cancer screening (10). Third, and just as important, participating in the BCCP might provide women the opportunity to connect and develop a continuity of care relationship with primary care providers. This factor is strongly and positively associated with screening mammography (11,12) and reduced breast cancer mortality (13), especially given the role of primary care providers in preventing treatment delays (14). Additional factors contributing to better survival in women with screening-detected breast cancer are breast cancer diagnosis at earlier stages of the disease and the fact that screening-detected tumors tend to be slower-growing than those detected otherwise (15). This survival advantage in screening-detected breast cancer patients over other breast cancer patients persists even after adjusting for tumor characteristics (15,16).

The objective of this study was to compare screening and cancer-related outcomes between 2 groups of Medicare beneficiaries: former BCCP participants and their low-income non-BCCP counterparts. We hypothesized that among Medicare beneficiaries with breast cancer, former BCCP participants would be more likely than their low-income non-BCCP counterparts to be diagnosed with cancer after screening, rather than after diagnostic (or no) mammogram, and more likely to have better treatment and survival outcomes.

## Methods

We used linked records from the Ohio Cancer Incidence Surveillance System (OCISS), the state’s BCCP data, Medicare enrollment and claims files, the US Census, and death certificate files. This study was approved by the Case Western Reserve University institutional review board (IRB); by the IRB of the Ohio Department of Health, which maintains the OCISS, BCCP, and death certificate files; and by the Centers for Medicare and Medicaid Services (CMS).

### Data sources and linkage

#### Ohio Cancer Incidence Surveillance System

Established in 1991, the OCISS captures data on incident cases of cancer diagnosed among Ohio residents. With the exception of carcinoma in situ of the cervix and nonmelanoma skin cancers, all other cancers are required by law to be reported to the OCISS. According to a report by the OCISS, its completeness for female breast cancer was on average 88% during the study period (personal communication, Holly Sobotka, MS, Ohio Department of Health, March 2015).

In addition to patient identifiers, the OCISS record has the following information: patient residence address (used for geocoding); patient demographics (age at time of diagnosis and race); date of diagnosis; anatomic cancer site; and the Surveillance, Epidemiology, and End Results (SEER) summary stage. Although the variables on tumor size, number of lymph nodes, and metastatic status are available through the OCISS, these variables have large proportions of missing values. Thus, we used the SEER summary stage rather than a more detailed stage classification.

In this study, the OCISS was used to identify women diagnosed with incident breast cancer from January 1, 2000, through June 30, 2009, and to retrieve the geocoded address of the patient, her demographics, date of cancer diagnosis, and tumor stage.

#### BCCP database

This database includes records for women served by Ohio’s BCCP since its inception in 1994. It comprises 3 files: the first file includes 1 record for each participant and has identifiers, which were used to link with data from the OCISS; the second file includes a record for each encounter; and the third file itemizes the procedures received at each encounter. From this database, we identified 1-time BCCP participants and repeat BCCP participants.

#### Medicare enrollment and claims files

The Master Beneficiary Summary File (MBSF) includes 1 record for each individual enrolled in Medicare in a given calendar year. In addition to patient demographics, the MBSF record has monthly variables to indicate beneficiaries’ participation in Part A, Part B, and managed care programs as well as dual Medicare–Medicaid enrollment status.

The Medicare Provider, Analysis, and Review (MedPAR) file includes records for hospital admissions. Each record indicates the date of service and *International Classification of Diseases, 9th Revision, Clinical Modification* (ICD-9-CM) diagnosis and procedure codes. The Outpatient and Carrier Standard Analytical Files (SAFs) include claims for services received in outpatient institutional and noninstitutional settings. Records from these files also indicate dates of services and ICD-9-CM diagnosis codes. The procedure codes are documented in Current Procedural Terminology, 4th Edition (CPT-4), or in the Healthcare Common Procedure Coding System.

These files, which were available to us for 2000 through 2009, were used to further define our study population and to identify comorbid conditions, nursing home status, and timing and receipt of cancer treatment.

#### US Census data

Using the geocoded addresses from the OCISS, we obtained data on income and educational attainment for the census tract and census block group levels. Census tracts were also used to determine whether a woman’s area of residence was in a Medically Underserved Area (MUA) or was part of a Medically Underserved Population (MUP), according to areas listed by the Health Resources and Service Administration and the Ohio Department of Health. The listings were obtained in March 2012.

#### Ohio death certificate data

The Ohio death certificate file includes a death certificate record for every deceased resident of Ohio. In addition to identifiers, the death certificate indicates the date and underlying cause of death. This file was used to analyze all-cause survival and cancer survival.

First we linked records from the OCISS with records in BCCP files, using patient identifiers (patient first and last name, social security number, and date of birth) and relying on a multistep deterministic matching algorithm, consistent with previous studies (17–19). Next, identifiers were sent to CMS for linkage with Medicare files.

### Study population

The study population included women with low incomes, residing in Ohio, aged 66 to 74, and diagnosed with incident invasive breast cancer during the study period (n = 14,769). Although the age of enrollment in Medicare is 65 years, we used 66 years as the lower age limit to allow for a 1-year look-back period to identify comorbidities and receipt of screening mammography. We set the upper age limit at 74 because the US Preventive Services Task Force does not recommend screening mammography beyond that age (20).

From this group, we selected 1) those who had participated in the BCCP program before becoming age-eligible for Medicare, and 2) low-income Medicare beneficiaries. To overcome the absence of income data for Medicare beneficiaries, we used the median household income at the census block group level (MHI-CB) as a proxy for individual income. Medicare beneficiaries were identified as low-income if the MHI-CB was below the 10th percentile, based on the distribution of this measure for women with breast cancer statewide during the study period.

To minimize the chances of having incomplete claims data, we limited our study population to those who were enrolled in Part A and Part B and received their care through the traditional fee-for-service system in the 12 months before and 6 months after cancer diagnosis. We also excluded women with unstaged or unknown-stage cancer. Our final study population included 513 women.

### Variables of interest

Our outcome variables were receipt of screening mammography in the previous year, advanced-stage cancer, time to treatment, receipt of standard treatment, and all-cause and cancer survival. Screening mammography was identified in claims indicating a CPT code of 76092, with no evidence in any of the patient’s claims files of a previous diagnosis of breast cancer (ICD-9 codes 174.xx or 233.0) or breast mass (ICD-9 code 611.72) in the previous year, or a mammogram (CPT codes 76090–76092) in the previous 11 months (21). Advanced-stage cancer was defined as regional-stage or distant-stage cancer at diagnosis, excluding cases that were unstaged or unknown-stage cancer. Time to treatment was defined as the time elapsed between date of diagnosis and receipt of the first cancer-directed treatment. Receipt of standard treatment was defined for local-stage disease as 1) mastectomy or 2) lumpectomy plus radiation therapy, and for regional-stage disease as the same treatment as for local-stage disease plus chemotherapy. All-cause survival and cancer survival were based on the time elapsed between date of cancer diagnosis and date of death and the underlying cause of death.

Our main independent variable was BCCP status (yes or no), defined as yes if a woman was identified in the OCISS, BCCP, and Medicare files. In addition, we created a variable to indicate whether a woman had participated in BCCP only once (defined as one-time participant) or multiple times (defined as repeat participant). Because of the small sample sizes of the one-time and repeat participants, we conducted the multivariable analyses after combining the 2 groups.

We grouped women according to selected demographic characteristics. We established 2 categories of age (66–69 y, 70–74 y) and 2 categories of race (African American and other); we combined the category for white with the categories for all other races/ethnicities because of the small number of cases who were not white or African American. We established 3 categories of marital status (married, not married, and unknown). Low educational attainment was defined as residing in a census block group in which the percentage of adults with a high school diploma was in the lowest 10th percentile, based on the distribution of this measure for women with breast cancer statewide. For place of residence, we created indicators of whether women resided in Appalachian/rural, metropolitan, or suburban counties and whether they resided in a census tract or county identified as an MUA or MUP. In some counties, an MUA was based on a service area, rather than a census tract; accordingly, we coded a county as MUA or MUP for all or an unknown part of the county. Thus, counties were categorized as none, part, or all/unknown MUA/MUP. Finally, to characterize the chronic disease burden and complexity of care for our study population, we created variables for 1) number of comorbidities (0, 1, or ≥2) identified in claims data for services received in the year before cancer diagnosis, using Elixhauser’s listing of conditions (22); 2) dual Medicare–Medicaid enrollment (yes or no), as identified from the monthly indicators in the Medicare enrollment file for the 12-month period before cancer diagnosis; and 3) residence in a nursing home (yes or no) in the 6 months before or after cancer diagnosis, using a validated claims-based algorithm (23).

### Analysis

In addition to descriptive analysis, we used multivariable regression analysis to evaluate the association between BCCP status and each of the outcomes, after adjusting for patient covariates. For dichotomous variables, we developed logistic regression models; for time-to-event models, we developed Cox proportional hazard models. We conducted 2 additional analyses: propensity score to adjust for potential selection bias and sensitivity analysis by income level.

The propensity scores, based on age, race, marital status, educational attainment, dual Medicare–Medicaid eligibility status, area of residence, residence in a MUA or MUP, number of comorbidities, and residence in nursing home, were included in the regression models in quintiles. For the cancer survival model, we combined the 4th and 5th quintiles to remedy for small numbers.

According to the BCCP, low income is defined as an income below 200% of the federal poverty level — a measure based on the number of people in a household. As noted earlier, however, we identified low-income women as those residing in block groups with MHI-CB in the lowest 10th percentile (24). We therefore conducted a sensitivity analysis by comparing outcomes among BCCP women with those of non-BCCP women at each of 2 income levels: MHI-CB in the lowest 5th percentile and MHI-CB in the lowest 15th percentile.

## Results

Our study population had 93 BCCP and 420 low-income non-BCCP women. Of the BCCP women, 57 (61%) were repeat participants. The BCCP group and non-BCCP group differed significantly in their sociodemographic distribution and place of residence (Table 1). The percentage of women aged 70 to 74 was significantly higher among non-BCCP women than among BCCP women (58.1% vs 30.1%). In addition, we found a lower percentage of African American women in the BCCP group (19.4% vs 39.8%); a lower percentage of women residing in areas of low educational attainment (small numbers and percentages masked in accordance with Centers for Medicare and Medicaid Services’ privacy rules); a lower percentage residing in MUA or MUP regions (66.7% vs 88.6%); and a lower percentage of women with 1 or more comorbid conditions (66.7% vs. 75.7%).

**Table 1:** Distribution of the Study Population Among BCCP Women and Low-Income^a^ Non-BCCP Women, Ohio, 2000–2009^b^

Characteristic	BCCP One-Time Users (N = 36)^c^	BCCP Repeat Users (N = 57)^c^	All BCCP Users (N = 93)^d^	Low-Income Non-BCCP Users, (N = 420)
**Age, y**				
66–69	66.7	71.9	69.9^e^	41.9
70–74	33.3	28.1	30.1	58.1
**Race**				
African American	—	—	19.4^e^	39.8
Other	—	—	80.6	60.2
**Marital status**				
Married	—	—	—	23.6
Not married	69.4	64.9	66.7	70.5
Unknown	—	—	—	6.0
**Educational attainment^f^**				
Low	—	—	—e	46.7
High	—	—	—	53.3
**Dual Medicare–Medicaid eligibility**				
No	50.0	40.4	44.1	49.5
Yes	50.0	59.6	55.9	50.5
**County of residence**				
Appalachian or rural	41.7	43.9	43.0^e^	21.2
Metropolitan	—	—	40.9	72.4
Suburban	—	—	16.1	6.4
**Residence in medically underserved area or population**				
None	—	—	—e	3.3
Part	72.2	63.2	66.7	88.6
All/unknown	—	—	—	8.1
**No. of comorbidities**				
0	—	38.6	33.3^g^	24.3
1	—	29.8	30.1	22.6
≥2	44.4	31.6	36.6	53.1
**Cancer stage at diagnosis**				
Local	58.3	75.4	68.8	67.9
Regional or distant	41.7	24.6	31.2	32.1
**Lives in nursing home**				
No	—	—	88.2	80.2
Yes	—	—	11.8	19.8
**Had screening mammography in year before cancer diagnosis**				
No	52.8	45.6	48.4^g^	61.2
Yes	47.2	54.4	51.6^g^	38.8
**Time to treatment initiation, days**				
Median (95% CI)	49 (23–117)	35 (22–50)	41 (28–53)	34 (30–40)
Mean (SD)	61.4 (7.9)	50.8 (6.1)	56.8 (5.2)	62.8 (3.3)
**Receipt of standard treatment^h^**				
No	38.2	20.4	27.3	21.6
Yes	61.8	79.6	72.7	78.4

Abbreviations: — , small cells (n < 11) were masked in accordance with the privacy rules of the Centers for Medicare and Medicaid Services, and an additional cell in the same column was also masked to prevent the reader from deriving the numbers; BCCP, National Breast and Cervical Cancer Early Detection Program; CI, confidence interval; SD, standard deviation.

a Medicare beneficiaries were identified as low income if the median household income in the residence census block group was below the 10th percentile, based on the distribution of median household incomes across the state for breast cancer patients during the study period.

b Values are expressed as percentage of total unless otherwise indicated.

c BCCP one-time users and BCCP repeat users were compared; no significant differences were found (*P* < .05).

d Superscripts in this column refer to the comparison of measures between all BCCP women and low-income non-BCCP women; all other comparisons, *P* < .05.

e *P* < .001.

f Low educational attainment defined as residing in a census block group in which the percentage of adults with a high school diploma was in the lowest 10th percentile, based on the distribution of adults with high school diplomas across the state for breast cancer patients during the study period.

g *P* = .02.

h Analysis of treatment limited to women diagnosed with local-stage or regional-stage cancer.

The percentage of women undergoing screening mammography in the year before cancer diagnosis was 51.6% in the BCCP group and 38.8% in the low-income non-BCCP group (*P* = .02). However, the proportion of women diagnosed with regional-stage or distant-stage disease was similar in the 2 groups (31.2% vs 32.1%), and it was lowest among repeat BCCP users (24.6%). Receipt of standard treatment was lower in the BCCP group than in the low-income non-BCCP group (72.7% vs 78.4%) and lowest among one-time BCCP users (61.8%). None of these differences were significant. Similarly, neither all-cause survival (log-rank χ^2^ = 1.2; *P* = .27) nor cancer survival (log-rank χ^2^ = 1.7; *P* = .19) differed significantly between the 2 groups.

Adjusting for potential confounders and propensity scores, we found that the BCCP group was significantly more likely than the low-income non-BCCP group to have had screening mammography (adjusted odds ratio [AOR], 1.77; 95% confidence interval [CI], 1.01–3.09) (Table 2). No significant differences were detected in any other outcomes (Table 2 and Table 3).

**Table 2:** Results From the Multivariable Analysis for Screening Mammography, Cancer Stage at Diagnosis, Time to Treatment Initiation, and Receipt of Standard Treatment, Adjusted for Propensity Scores^a^, Ohio, 2000–2009

Characteristic	Screening Mammography	Advanced-Stage Cancer at Diagnosis	Time to Treatment Initiation	Receipt of Standard Treatment
**Breast and Cervical Cancer Early Detection Program**				
Yes	1.77 (1.01–3.09)^b^	1.04 (0.57–1.87)	0.90 (0.67–1.23)	0.69 (0.36–1.33)
No	1 [Reference]	1 [Reference]	1 [Reference]	1 [Reference]
**Age, y**				
66–69	1 [Reference]	1 [Reference]	1 [Reference]	1 [Reference]
70–74	1.10 (0.67–1.81)	1.01 (0.61–1.69)	0.94 (0.72–1.23)	0.66 (0.35–1.24)
**Race**				
African American	1.00 (0.64–1.56)	1.13 (0.71–1.78)	0.78 (0.61–0.99)^c^	0.87 (0.50–1.51)
Other	1 [Reference]	1 [Reference]	1 [Reference]	1 [Reference]
**Marital status**				
Married	1 [Reference]	1 [Reference]	1 [Reference]	1 [Reference]
Not married	1.20 (0.75–1.92)	1.00 (0.61–1.63)	0.82 (0.64–1.05)	1.13 (0.65–1.97)
Unknown	0.63 (0.25–1.54)	0.51 (0.20–1.34)	0.69 (0.43–1.10)	1.35 (0.43–4.25)
**Educational attainment^d^**				
Low	0.53 (0.30–0.93)^e^	1.04 (0.57–1.89)	1.03 (0.76–1.40)	0.77 (0.39–1.53)
High	1 [Reference]	1 [Reference]	1 [Reference]	1 [Reference]
**Dual Medicare–Medicaid eligibility**				
No	1 [Reference]	1 [Reference]	1 [Reference]	1 [Reference]
Yes	0.87 (0.56–1.36)	1.45 (0.91–2.31)	0.91 (0.72–1.16)	1.36 (0.79–2.35)
**County of residence**				
Appalachian or rural	2.55 (1.33–4.87)^f^	0.94 (0.47–1.89)	0.98 (0.68–1.41)	0.90 (0.42–1.92)
Metropolitan	1 [Reference]	1 [Reference]	1 [Reference]	1 [Reference]
Suburban	2.01 (0.91–4.46)	1.43 (0.63–3.26)	1.18 (0.78–1.78)	0.65 (0.27–1.61)
**Residence in medically underserved area or population**				
None	1 [Reference]	1 [Reference]	1 [Reference]	1 [Reference]
Part	0.83 (0.28–2.47)	1.43 (0.42–4.89)	0.88 (0.50–1.55)	0.53 (0.13–2.12)
All/unknown	0.37 (0.10–1.32)	2.06 (0.50–8.47)	0.81 (0.42–1.56)	0.67 (0.14–3.36)
**No. of comorbidities**				
0	1 [Reference]	1 [Reference]	1 [Reference]	1 [Reference]
1	0.89 (0.52–1.51)	0.83 (0.48–1.44)	1.11 (0.84–1.47)	0.54 (0.28–1.05)
≥2	0.64 (0.38–1.09)	0.55 (0.32–0.95)^g^	0.81 (0.61–1.08)	0.52 (0.27–1.03)
**Cancer stage at diagnosis**				
Local	1 [Reference]	—	1 [Reference]	1 [Reference]
Regional	0.52 (0.33–0.81)^h^	—	1.08 (0.85–1.38)	0.25 (0.15–0.42)^e^
Distant	0.43 (0.15–1.25)	—	—	—
**Lives in nursing home**				
No	1 [Reference]	1 [Reference]	1 [Reference]	1 [Reference]
Yes	0.47 (0.26–0.83)^i^	1.90 (1.13–3.22)^j^	1.08 (0.80–1.46)	1.04 (0.51–2.11)
**Had screening mammography in year before cancer diagnosis**				
No	—	1 [Reference]	1 [Reference]	1 [Reference]
Yes	—	0.51 (0.34–0.79)^k^	0.92 (0.74–1.15)	0.50 (0.31–0.82)^l^
**Receipt of standard treatment^m^**				
No	—	—	1 [Reference]	—
Yes	—	—	2.50 (1.90–3.29)^e^	—

a Except for the outcome of time to treatment initiation, all values are adjusted odds ratio (95% confidence interval). Values for the outcome of time to treatment initiation are adjusted hazard ratio (95% confidence interval). Values for propensity scores (in quintiles) were not shown because they have no bearing on the interpretation of the findings. *P* values are for analysis of maximum likelihood estimates; unless otherwise indicated, odds ratios and hazard ratios are not significant at *P* < .05.

b *P* = .045.

c *P* = .04.

d Low educational attainment defined as residing in a census block group in which the percentage of adults with a high school diploma was in the lowest 10th percentile, based on the distribution of this measure for women with breast cancer statewide

e *P* < .001.

f *P* = .005.

g *P* = .03.

h *P* = .004.

i *P* = .01.

j *P* = .02.

k *P* = .002.

l *P* = .006.

m Analysis limited to patients diagnosed with local-stage or regional-stage cancer.

**Table 3:** Results From the Multivariable Survival Analysis, Adjusted for Propensity Scores^a^, Ohio, 2000–2009

Characteristic	All-Cause Survival	Cancer Survival^b^
**Breast and Cervical Cancer Early Detection Program**		
Yes	1.11 (0.59–2.06)	1.13 (0.38–3.33)
No	1 [Reference]	1 [Reference]
**Age, y**		
66–69	1 [Reference]	—
70–74	0.92 (0.59–1.43)	—
**Race**		
African American	0.99 (0.66–1.47)	—
Other	1 [Reference]	—
**Marital status**		
Married	1 [Reference]	—
Not married	1.34 (0.82–2.20)	—
Unknown	0.96 (0.41–2.24)	—
**Educational attainment^c^**		
Low	0.90 (0.56–1.45)	—
High	1 [Reference]	—
**Dual Medicare–Medicaid eligibility**		
No	1 [Reference]	—
Yes	1.33 (0.89–1.99)	—
**County of residence**		
Appalachian or rural	0.69 (0.34–1.39)	—
Metropolitan	1 [Reference]	—
Suburban	0.99 (0.46–2.14)	—
**Residence in medically underserved area or population**		
None	1 [Reference]	—
Part	0.29 (0.10–0.82)^a^	—
All/unknown	0.34 (0.10–1.17)	—
**No. of comorbidities**		
0	1.00 [Reference]	—
1	1.23 (0.67–2.28)	—
≥2	1.82 (1.09–3.04)^a^	—
**Cancer stage at diagnosis**		
Local	1.00 [Reference]	1.00 [Reference]
Regional	1.49 (1.01–2.19)^e^	3.59 (1.90–6.76)^f^
Distant	—	—
**Lives in nursing home**		
No	1.00 [Reference]	—
Yes	2.04 (1.36–3.06)^f^	—
**Had screening mammography in year before cancer diagnosis**		
No	1.00 [Reference]	—
Yes	0.68 (0.45–1.02)	—
**Receipt of standard treatment**		
No	1.00 [Reference]	—
Yes	0.63 (0.39–1.02)	—

a All values are adjusted hazard ratio (95% confidence interval). Values for propensity scores (in quintiles) are not shown, because they have no bearing on the interpretation of the findings. *P* values are for analysis of maximum likelihood estimates; unless otherwise indicated, odds ratios are not significant at *P* < .05. Analysis limited to patients diagnosed with local-stage or regional-stage cancer.

b Given the small number of events, we present the adjusted hazard ratio and 95% confidence interval from the reduced model, which included only the BCCP variable, propensity scores, and cancer stage (the only independent variable that was significant at *P* < .05).

c Low educational attainment defined as residing in a census block group in which the percentage of adults with a high school diploma was in the lowest 10th percentile, based on the distribution of this measure for women with breast cancer statewide.

d *P* = .02.

e *P* = .046.

f *P* < .001.

The sensitivity analysis by income level yielded 1 noteworthy difference: when we compared outcomes between BCCP women and non-BCCP women residing in census block groups with MHI-CB in the lowest 5th percentile, we found no significant difference in the likelihood to have had screening mammography (AOR, 1.23; 95% CI, 0.63–2.40); however, this may have been due to a small sample size. In contrast, when comparing outcomes between BCCP women and non-BCCP women residing in census block groups with incomes in the lowest 15th percentile, BCCP women were nearly twice as likely to have undergone screening mammography (AOR, 1.90; 95% CI, 1.16–3.12).

## Discussion

This study compared breast cancer outcomes between Medicare beneficiaries with a history of participation in the BCCP and their low-income non-BCCP counterparts. The findings indicated that compared with low-income non-BCCP Medicare beneficiaries, former BCCP women were significantly more likely to have undergone screening mammography before cancer diagnosis. The results pertaining to breast cancer outcomes were mixed, although none of the differences were significant.

To our knowledge, this is the first study to document the lasting effects of participation in the BCCP among women who become age-eligible and transitioned to Medicare. The differentials in the outcomes of interest, or lack thereof, provide a glimpse of what we might observe after the implementation of the Affordable Care Act (ACA), when many uninsured women receive insurance coverage. Nonetheless, to a great extent, these findings, especially the greater likelihood of former BCCP participants to undergo screening mammography, reflect important differences in health care–seeking behaviors between former BCCP participants and their low-income non-BCCP counterparts. However, reasons why better screening did not translate into early-stage diagnosis and receipt of standard treatment remain to be explored.

Our study has several limitations. First, the lack of significant differences probably resulted from a small sample size, especially for BCCP participants, despite pooling nearly 10 years of data. In a follow-up study, it would be interesting to expand the study population by including data from multiple states. Second, low-income women in our comparison group were identified on the basis of MHI-CB rather than at the individual level; however, such measures have been deemed adequate to evaluate socioeconomic inequalities (25). Furthermore, our criterion for defining low-income women (those residing in census block groups with an MHI-CB in the lowest 10th percentile) was somewhat arbitrary, although consistent with a previous study (24). To address this limitation, we conducted sensitivity analyses by identifying comparison groups at 2 income thresholds — one with MHI-CB at the lowest 5th percentile and the other with MHI-CB at the lowest 15th percentile — and we found some differences in screening mammography. We also noted some differences in cancer stage at diagnosis, although these differences were not significant. Third, because this study was not randomized, there may have been selection bias due to inherent differences between former BCCP women and non-BCCP women. We addressed this limitation with the propensity score approach in our analysis.

Despite these weaknesses, our study informs the discussion on the contribution of the BCCP to the improvement of breast cancer outcomes in low-income women, especially in the context of Medicaid expansion and the implementation of the ACA. While the new policies will likely improve access to care, it will be important to determine whether, through an organized rather than an opportunistic approach to screening (26,27), the BCCP may offer a venue to improve breast and cervical cancer screening above and beyond the improvements that might be achieved with the implementation of Medicaid expansion and the ACA alone, especially given low reimbursement rates and providers’ reluctance to serve Medicaid patients. Indeed, while an organized approach to screening relies on policy and an adequate call-recall system, the opportunistic approach will depend on individual-related and provider-related factors, such as knowledge and behavior. Future studies weighing the benefits of Medicaid expansion and the ACA versus the benefits of the BCCP need to account for these factors.
