# Erratum, Vol. 12, July 23 Release

**DOI:** 10.5888/pcd12.140491e

**Published:** 2015-09-24

In the article “Lasting Effects of the Breast and Cervical Cancer Early Detection Program on Breast Cancer Detection and Outcomes, Ohio, 2000–2009,” an error was made in defining standard treatment among women who had a diagnosis of local-stage cancer; the definition failed to account for radiation therapy. The authors reran their analyses, and although the conclusions of the study did not change, the raw percentages of women receiving standard treatment in the various subgroups did, and so did some of the results from the multivariable analysis. Changes were made to Table 1 (last 2 rows), Table 2 (last 2 columns), and Table 3 (middle column). In addition, the percentages in the following sentence found in the second paragraph of the Results section were changed to “Receipt of standard treatment was lower in the BCCP group than in the low-income non-BCCP group (70.5% vs 71.4%) and lowest among one-time BCCP users (58.8%).”

The changes were made to our website on August 26, 2015, and appear online at http://www.cdc.gov/pcd/issues/2015/14_0491.htm. We regret any inconvenience or confusion this error may have caused.

